# Construction of phylogenetic trees by kernel-based comparative analysis of metabolic networks

**DOI:** 10.1186/1471-2105-7-284

**Published:** 2006-06-06

**Authors:** S June Oh, Je-Gun Joung, Jeong-Ho Chang, Byoung-Tak Zhang

**Affiliations:** 1 Department of Pharmacology, Inje University College of Medicine, Busan, 614-735, Korea; 2 Center for Bioinformation Technology, Seoul National University, Seoul, 151-742, Korea; 3 Graduate Program in Bioinformatics, Seoul National University, Seoul, 151-742, Korea; 4 Biointelligence Laboratory, School of Computer Sci. and Eng., Seoul National University, Seoul, 151-742, Korea

## Abstract

**Background:**

To infer the tree of life requires knowledge of the common characteristics of each species descended from a common ancestor as the measuring criteria and a method to calculate the distance between the resulting values of each measure. Conventional phylogenetic analysis based on genomic sequences provides information about the genetic relationships between different organisms. In contrast, comparative analysis of metabolic pathways in different organisms can yield insights into their functional relationships under different physiological conditions. However, evaluating the similarities or differences between metabolic networks is a computationally challenging problem, and systematic methods of doing this are desirable. Here we introduce a graph-kernel method for computing the similarity between metabolic networks in polynomial time, and use it to profile metabolic pathways and to construct phylogenetic trees.

**Results:**

To compare the structures of metabolic networks in organisms, we adopted the exponential graph kernel, which is a kernel-based approach with a labeled graph that includes a label matrix and an adjacency matrix. To construct the phylogenetic trees, we used an unweighted pair-group method with arithmetic mean, i.e., a hierarchical clustering algorithm. We applied the kernel-based network profiling method in a comparative analysis of nine carbohydrate metabolic networks from 81 biological species encompassing Archaea, Eukaryota, and Eubacteria. The resulting phylogenetic hierarchies generally support the tripartite scheme of three domains rather than the two domains of prokaryotes and eukaryotes.

**Conclusion:**

By combining the kernel machines with metabolic information, the method infers the context of biosphere development that covers physiological events required for adaptation by genetic reconstruction. The results show that one may obtain a global view of the tree of life by comparing the metabolic pathway structures using meta-level information rather than sequence information. This method may yield further information about biological evolution, such as the history of horizontal transfer of each gene, by studying the detailed structure of the phylogenetic tree constructed by the kernel-based method.

## Background

The availability of pathway databases such as Kyoto Encyclopedia of Genes and Genomes (KEGG), What is there? (WIT3), PathDB, and MetaCyc opens up various new possibilities for comparative analysis. In particular, information about metabolic pathways in different organisms yields important information about their evolution and offers a complementary approach to phylogenetic analysis. Here we present a comparative metabolomic approach to constructing phylogenetic trees that uses physiological functions of the organisms by computing the structural similarity of metabolic networks. The consideration of metabolic components complements the conventional approaches to phylogeny based on genome sequences. Recognizing the similarities and differences in metabolic functions between species may provide insights into other applications in biotechnology, ecology, and evolutionary studies. Several researchers have attempted to rebuild evolutionary history by comparing ribosomal RNA sequences [1], by phylogenomics [2], or by comparing whole genomes to overcome the limitations of the gene-sequence analyses [3-5].

Several recent studies have extended conventional phylogenetic analysis to incorporate metabolic pathway information. Forst and Schulten [6,7] presented one of the earliest approaches to extend the conventional sequence comparison and phylogenetic analysis of individual enzymes to metabolic networks. They also presented a method to calculate distances between metabolic networks based on sequence information of the biomolecules involved and information about the corresponding reaction networks. Dandekar *et al*. (1999) combined strategies in a systematic comparison of the enzymes and corresponding sequence information of the glycolytic pathway [8]. Other approaches involving the reconstructed phylogenies from gene-order data have been based on simulating genome evolution [9], and studying the genome evolution resulting from the metabolic adaptation of the organism to the surrounding environment. Liao *et al*. (2002) presented a method to group organisms by comparing the profiles of metabolic pathways, where the profiling was based simply on binary attributes (e.g., by denoting the presence or absence of pathways in the organisms) [10].

Whereas the previous approaches incorporated information about the additional metabolic pathways, systematic methods to calculate the similarities between metabolic networks are lacking or contain gaps in some of the biological assumptions. In this paper, we introduce the concept of graph kernels to calculate the similarities between two different network structures. The graph kernel-based approach can compute more efficiently the similarity of two graph structures by the kernel function that can extract important features from the graph. Our approach contrasts with that of Forst and Schulten [6,7] in that the graph kernel calculates the distance based on the network level instead of on its sequence information on the biomolecules involved.

In their comparative analysis of metabolic pathways, Heymans and Singh [11] showed that phylogenetic trees could be made from the graph similarities of metabolic networks. They applied a distance measure between metabolic graphs of the glycolytic pathway and the citric acid cycle from 16 organisms. However, some of their data on phylogenetic inference did not correspond entirely with the conventional taxonomy and did not provide a global view of the specialization of species according to the scale of analyzed species and metabolic pathways.

Several more recent attempts have reconstructed genome trees using different formalisms such as gene ordering [12,13], measuring gene contents [3,14], comparing sequence similarities [15,16], comparing proteome strings [17], and phylogenomics [18,19]. All are based on the principle of genome sequences, but none has applied the concepts of effectible physiology to the phylogenetic analyses. We report on our comparative results and discuss our findings.

## Results and discussion

**Figure 1:**
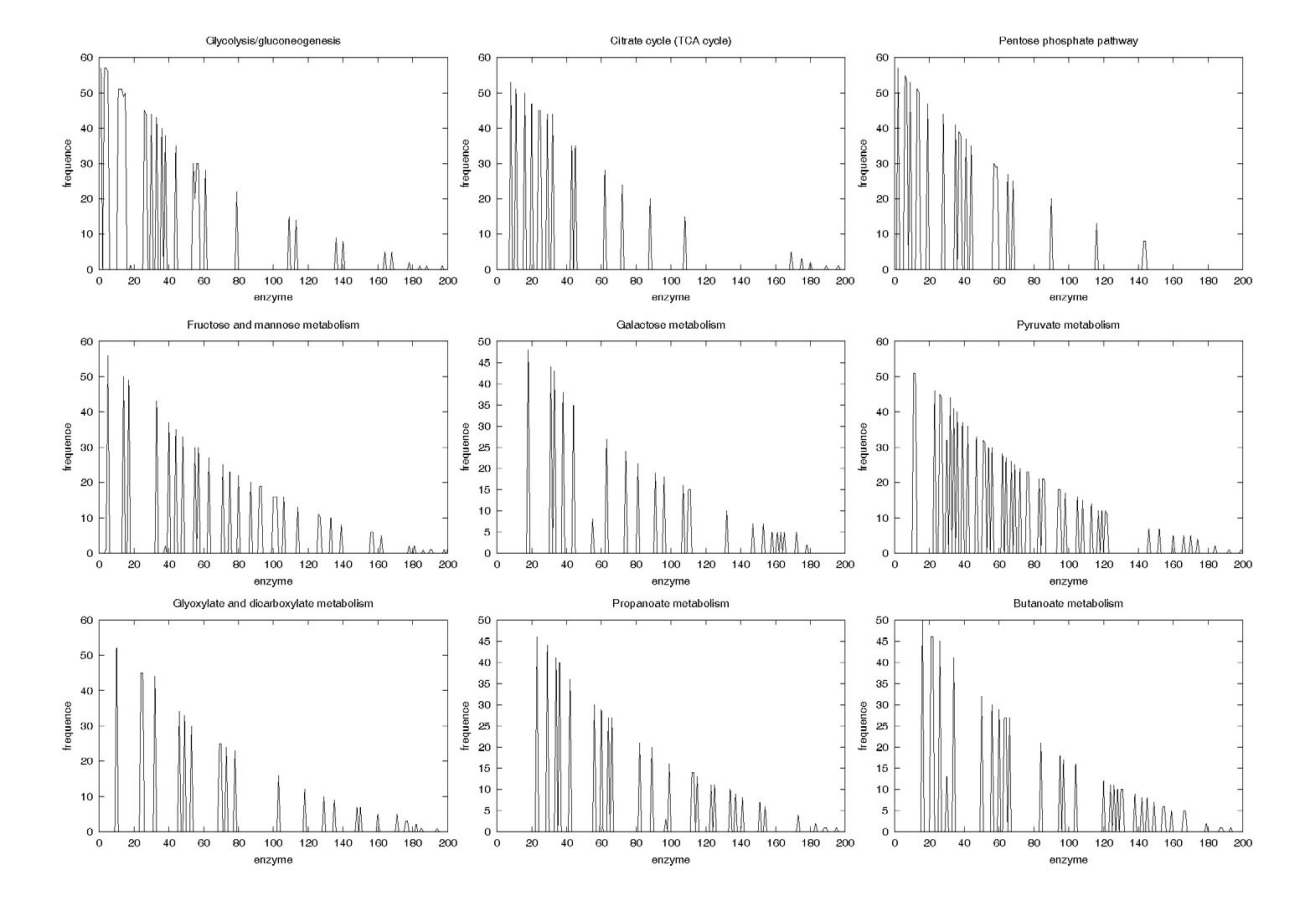
The distribution of enzymes in nine reference pathways. The *x *axis is the index of the maximum value of the order sorted by the frequency of enzyme over all pathways. All plots showed similar distributions.

We chose nine pathways of carbohydrate metabolism and 81 species to perform a comparative analysis of metabolic networks that satisfied the most abundant dataset from KEGG (Table 1, 2 and 3). Our sample comprised 13 species of Archaea, eight species of Eukaryota, and 60 species of Eubacteria. The central pathways of metabolism include the glycolytic and pentose phosphate pathways, and the citric acid cycle, which generate biological energy and form the metabolic precursors essential for almost all living cells. To validate the data, we investigated the distribution of each enzyme in the nine pathways. Several enzymes appear at a high frequency in all species, and this frequency decreases rather exponentially as the value of x-axis increases (Figure 1), a phenomenon we observed in all pathways studied. Because the characteristics of the enzyme distribution did not differ, all pathways can be used in the phylogenetic analysis. We observed no obvious tendency of shift or deviation of the distributions from the overall pattern of pathways.

The phylogeny took two directions: conventional taxonomy that focused on the morphological and physiological features to classify species, and a numerical taxonomy that stressed the historical changes in biological sequences. Phylogenies based on the ribosomal RNA molecules led to the proposal of a new tripartite scheme of three domains: Bacteria, Archaea, and Eukarya [20]. Although each approach is feasible on its own, it cannot provide a holistic view of the organism. Current phylogenetic studies indicate that horizontal gene transfer may have played a vital role in the evolution of major lineages [21]. Lake and Moore [22] also noted the pitfalls of comparative genomics based on molecular sequences. Our kernel-based method provides an alternative to the inference of an evolutionary scenario and allows for a higher-level comparison of the phylogenetic trees by measuring the distances between pathways using metabolic network data to infer an evolutionary scenario.

### Consistency with conventional taxonomy

**Figure 2:**
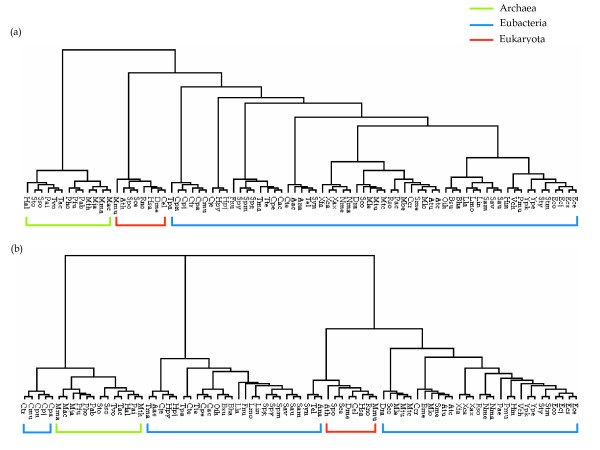
Comparison of phylogenetic trees constructed using (a) the kernel-based method and (b) the alignments of the following two enzyme sequences, together: phosphoglycerate kinase (EC 2.7.2.3) and phosphopyruvate hydratase (EC 4.2.1.11). The amino acid sequences of corresponding enzymes were retrieved from the GenBank database and analyzed by CLUSTAL with default parameters. The resultant trees were viewed with the TREEVIEW program [43].

**Figure 3:**
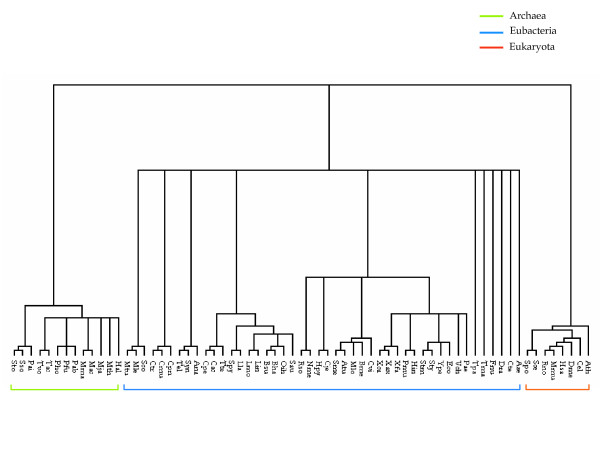
Current classification of biological taxonomy. The tree was reconstructed from part of the data in the NCBI (National Center for Biotechnology Information) Taxonomy [44] and viewed with the TREEVIEW program [43].

Figure 2 compares the phylogenetic tree constructed by the kernel method using (a) metabolic networks, and (b) by the sequence-based analysis of phosphoglycerate kinase and phosphopyruvate hydratase. The overall tree structure appears to be consistent with the current classification of the three domains, Eubacteria, Archaea, and Eukaryota (Figure 3). In the domain of Archaea, methanogens such as *Methanosarcina, Methanococcus*, and *Methanobacterium *are clustered in the early branch of *Pyrococcus*. Five thermophiles are also clustered in the same branch with a halophile, *Halobacterium*. The structure of collective carbohydrate pathways from *Arabidopsis thaliana *has the smallest distance from yeasts (Figure 2). As shown in Figure 2(a), the kernel-based method branches at the levels of family (*Enterobacteriaceae*), order (*Bacillales*), and class (*Gammaproteobacterium*) according to similar metabolic network structures. Figures 2(a) and 3 show that the domain of Archaea corresponded largely to that of conventional taxonomy ; NCBI Taxonomy Browser). In Eukaryota, only the mouse deviated from the taxonomy consensus. Typically, the domain of Archaea always formed a single cluster in every case in our experiments, suggesting that the domain of Archaea has peculiar characteristics, at least in the carbohydrate metabolic networks. We conclude that the metabolic structure in Archaea is distinct from that of the other two domains.

Figure 2(a) shows that archaeal metabolic networks are more closely related to the eukaryotic networks than with the eubacterial networks. This corresponds with a comparison of the information-transfer pathways and pathway-level organization between two domains [23], whereas eukaryotic metabolic enzymes are primarily of bacterial origin [24].

### Inferring hidden order by network clustering

The conventional sequence-based analysis passes over or does not embrace the discordant evolution of each species or the horizontal gene transfer [25]. Our method can cope with this limitation by taking into account the structural features of individual metabolic networks. The disagreement between the molecular sequence data of operational genes and the rRNA tree suggests that different genes have different evolutionary histories [26,27]. To address this problem, Li studied the mitochondrial genomes in relation to the problem of whole-genome phylogeny, where evolutionary events, such as genetic rearrangements that include gene transfer from the exterior, make genome alignments difficult [28].

To compare the kernel-based comparative analysis of metabolic networks to the sequence-based phylogenetic analysis, we analyzed two enzyme sequences that participate in carbohydrate metabolism in all 81 species together using a multiple sequence alignment (Figure 2(b)). In the resulting phylogenetic tree, Archaea and Eukaryota are clustered at each terminal; however, short-distance neighboring node members belong to fairly distant taxonomic groups. The overall structure of the tree eventually becomes remote from not only that of the kernel-based method (Figure 2(a)), but also from that of current taxonomy (Figure 3). Although the phylogenetic tree constructed from the multiple alignment of two enzyme sequences shows a few unusual characteristics, our approach provides a good solution. The cluster mainly comprised archaeal species including the bacterial members *Chlamydia *and *Chlamydophila*, and had long branches at the root of the tree. Three eubacterial members (Ana, Tel, and Syn) are more closely related to Eukaryota than Eubacteria. Moreover, eubacterial groups are separated over the topology, and the Eukaryota are inserted between them (Figure 2(b)).

This result shows an example of the sequence-based phylogenetic approach when we intend to perform phylogenetic analyses for as many species as possible. The sequence-based phylogenetic analysis can fail to precisely represent evolutionary history without an analysis using a set of whole sequences. Unfortunately, analyses using whole sequences require massive computing power and are highly complex. A sequence-based phylogenetic analysis can still be limited to cover a number of species, although alternative approaches exist. However, our method can easily solve this problem by utilizing given resources. In order to measure the quality of our constructed trees, we compared the phylogenetic tree based on our graph kernel method with that of Heymans and Singh [11] in terms of their similarity to conventional taxonomy. We used a software tool, 'Cousins' [31], which compares two alternative phylogenetic trees based on common cousin pairs in the trees. The comparison by cousin pairs is said to more focus on local similarity between two trees because it evaluates the similarity based on a cousin pair within a certain degree. Table 4 shows the similarity score of our kernel-based method in comparison with [11] for the glycolysis pathways of 65 organisms. Our method shows a better result in terms of the similarity score with the conventional NCBI taxonomy.

In this paper, we intended to present a meta-level analysis of biological systems to construct a unitary phylogenetic tree that could be used to interpret the context of biological evolution. Our results suggest that the phylogenetic analysis with submetabolic network information might also allow us to infer horizontal or lateral gene transfer. Our results also support the tripartite scheme of the three domains, Bacteria, Archaea and Eukaryota [20].

Comparing the pathogenic bacterial genomes by focusing on the pathways of bacterial and eukaryotic aminoacyl-tRNA synthesis showed that this pathway is uniquely prokaryotic/archaeal and that it is found widely among the pathogenic bacteria. This suggests that members of this pathway can be used as targets for novel antimicrobial drugs [32]. Metabolic analysis of pathogenic organisms may play a critical role in the selective treatment or prevention of diseases caused by these organisms by using this innovative concept to develop new drugs.

## Conclusion

Biological classification, taxonomy, and systematics are the profound themes in biology. Using phylogeny in evolutionary classification implies functional and morphological innovation, adaptive range, parallelism, and convergence. We have used a method based on the graph kernel to compare information on each metabolic network including cardinality, distance, and topology relating to metabolic networks as a type of undirected graph. Our results showed that our approach has potential in the macroscopic analysis of phylogenetic relationships among organisms in relation to horizontal gene transfer. To obtain information about each causal mechanism in the context of a similar phenotype, one should first analyze the phenomena at the level of a protein network. The analysis of a metabolic network is an example of this type of analysis. Biological entities that interact with the environment and eventually influence adaptation are a function of the activity of proteins and other bioactive molecules rather than gene order or genetic history.

The overall structure of the phylogenetic tree constructed from our experiments supports the tripartite scheme of the three domains Archaea, Eubacteria, and Eukaryota as described in an early report of Woese *et al*. [20]. The structures of metabolic pathway deduced from Archaea are more similar to those from Eukaryota than to those from Eubacteria. This agrees with the rooted universal tree of life [33,34] and the tree of life [35,36]. The metabolic network structures of organisms reflect their functional relationship with the environment, and the similarity might provide a measure of the organism's physiological functions. The trajectory of an organism's adaptation can be explained using the structure of its metabolic contents. Our approach can be extended to more organisms and applied to other types of biomolecular interactions, such as physical protein interactions in regulatory networks, to provide a basis for understanding the functional relationships between biological networks in different organisms.

## Methods

**Figure 4:**
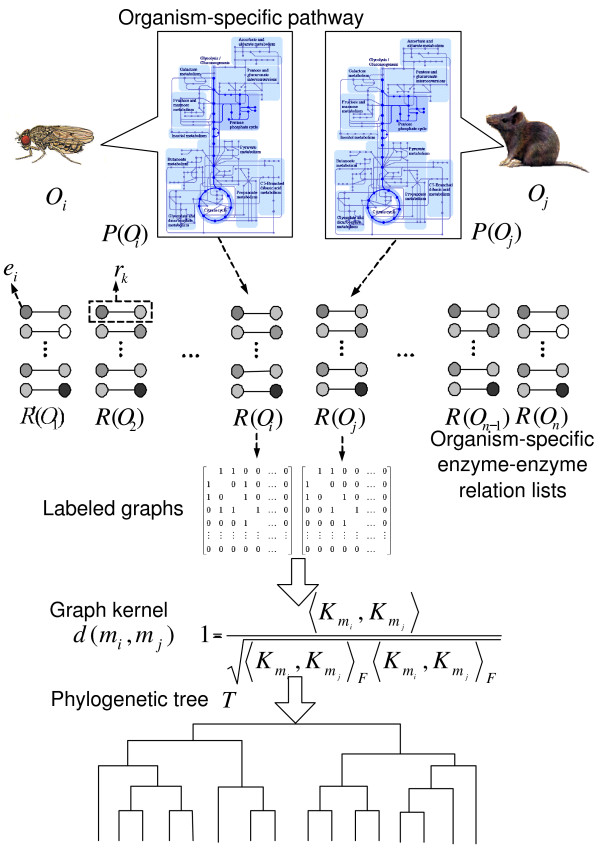
The procedure for data processing for phylogenetic tree construction from the metabolic networks.

We attempt to cluster organisms by comparing sets of metabolic pathways. Our basic assumption is that different species exhibit overlapping components of metabolic pathways. To construct phylogenetic trees, the features of the organism-specific pathways are automatically extracted by considering the reference pathway. Here, the features represent the connection information between two enzymes. Figure 4 summarizes the procedure for the phylogenetic clustering of organisms by metabolic pathways using four steps:

- Step 1: Build the enzyme-enzyme relation lists.

- Step 2: Convert the lists to graph structures.

- Step 3: Compute similarity by graph kernels.

- Step 4: Build the phylogenetic trees.

**Figure 5:**
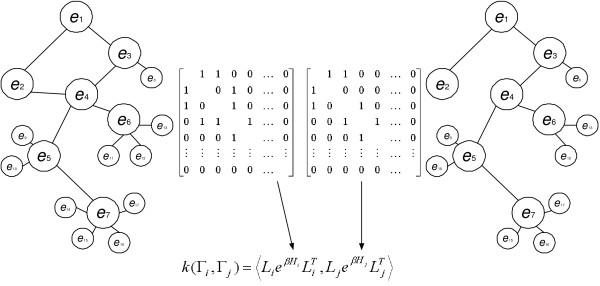
The simple concept for computing the similarity between two metabolic networks using the kernel method.

We evaluated this method using known experimental data on a collection of nine metabolic pathways from 81 representative organisms. Figure 5 shows the simple concept used to compute the distance between two metabolic networks. The resulting phylogenetic trees were cross-compared for consistency with existing methods to analyze phylogenies. The next section describes the datasets collected and the preparation methods, followed by the definition of graph kernels and their use in comparing and clustering metabolic networks for phylogenetic analysis.

### Data preparation

#### Dataset

We chose the KEGG database [37] as the resource for previous phylogenetic analysis. KEGG provides both an online map of pathways and the ability to focus on metabolic reactions in specific organisms. Each reaction may be uni- or bidirectional.

#### Representation of organisms

Let *O *= {*O*_1_,..., *O*_*N*_} be a set of *N *organisms and *P *= {*P*_1_,..., *P*_*M*_} be a set of *M *reference pathways. Here a reference pathway contains all known alternatives of reaction paths. The set of organism-specific pathways is defined as *P' *= {P′1
 MathType@MTEF@5@5@+=feaafiart1ev1aaatCvAUfKttLearuWrP9MDH5MBPbIqV92AaeXatLxBI9gBaebbnrfifHhDYfgasaacH8akY=wiFfYdH8Gipec8Eeeu0xXdbba9frFj0=OqFfea0dXdd9vqai=hGuQ8kuc9pgc9s8qqaq=dirpe0xb9q8qiLsFr0=vr0=vr0dc8meaabaqaciaacaGaaeqabaqabeGadaaakeaacuWGqbaugaqbamaaBaaaleaacqaIXaqmaeqaaaaa@2EFD@,...,P′M
 MathType@MTEF@5@5@+=feaafiart1ev1aaatCvAUfKttLearuWrP9MDH5MBPbIqV92AaeXatLxBI9gBaebbnrfifHhDYfgasaacH8akY=wiFfYdH8Gipec8Eeeu0xXdbba9frFj0=OqFfea0dXdd9vqai=hGuQ8kuc9pgc9s8qqaq=dirpe0xb9q8qiLsFr0=vr0=vr0dc8meaabaqaciaacaGaaeqabaqabeGadaaakeaacuWGqbaugaqbamaaBaaaleaacqWGnbqtaeqaaaaa@2F30@}, which contains organism-specific reactions. If we define a set of enzyme-enzyme relations as *R *= {*r*_1_,...,*r*_*K*_}, then a subset of *R *constitutes *P*_*j *_or P′j
 MathType@MTEF@5@5@+=feaafiart1ev1aaatCvAUfKttLearuWrP9MDH5MBPbIqV92AaeXatLxBI9gBaebbnrfifHhDYfgasaacH8akY=wiFfYdH8Gipec8Eeeu0xXdbba9frFj0=OqFfea0dXdd9vqai=hGuQ8kuc9pgc9s8qqaq=dirpe0xb9q8qiLsFr0=vr0=vr0dc8meaabaqaciaacaGaaeqabaqabeGadaaakeaacuWGqbaugaqbamaaBaaaleaacqWGQbGAaeqaaaaa@2F6A@ (1 ≤ *j *≤ *M*). Here, *r*_*k *_(1 ≤ *k *≤ *K*) is a pair of enzymes {*e*_*u*_, *e*_*v*_}, which means that *e*_*u *_directly connects with *e*_*v*_. The specific organism *O*_*i *_(1 ≤ *i *≤ *N*) contains a set of pathways *P*, defined as *P'*(*O*_*i*_) and including a subset of *R *for the specific organism, *R'*(*O*_*i*_).

#### Enzyme-enzyme relation lists of organisms

The pathways provided in KEGG are visualized on manually drawn pathway maps or XML-based graphics. To construct enzyme-enzyme relation lists, we used information about chemical compounds and chemical reactions contained in the LIGAND database [38]. The LIGAND database provides detailed molecular information about one type of the generalized protein-protein interaction, namely, the enzyme-enzyme relation. LIGAND is a composite database of ENZYME and COMPOUND. The ENZYME section contains information about enzymatic reactions and enzyme molecules, and the COMPOUND section contains more than 6,000 chemical compounds. The enzyme-enzyme relationship can be extracted from information about enzymes contained in the COMPOUND entries. We automatically extracted information about enzymes of a specific organism from the ENZYME section.

The enzyme-enzyme relationships of a specific organism were extracted from the enzyme-enzyme relation list. If two enzymes of *r*_*k *_in an enzyme-enzyme relation list existed in the enzyme list of a specific organism, we inserted *r*_*k *_into *R'*(*O*_*i*_). *P'*(*O*_*i*_) can be constructed by *R'*(*O*_*i*_).

### Data analysis

#### Metabolic networks as labeled graphs

Our approach to estimate the distance between two metabolic networks is based on the graph comparison. Using the relation list of enzymes, the metabolic network of each organism is represented by a labeled graph Γ = (V
 MathType@MTEF@5@5@+=feaafiart1ev1aaatCvAUfKttLearuWrP9MDH5MBPbIqV92AaeXatLxBI9gBamrtHrhAL1wy0L2yHvtyaeHbnfgDOvwBHrxAJfwnaebbnrfifHhDYfgasaacH8akY=wiFfYdH8Gipec8Eeeu0xXdbba9frFj0=OqFfea0dXdd9vqai=hGuQ8kuc9pgc9s8qqaq=dirpe0xb9q8qiLsFr0=vr0=vr0dc8meaabaqaciaacaGaaeqabaWaaeGaeaaakeaaimaacqWFveVvaaa@384B@, ℰ
 MathType@MTEF@5@5@+=feaafiart1ev1aaatCvAUfKttLearuWrP9MDH5MBPbIqV92AaeXatLxBI9gBamrtHrhAL1wy0L2yHvtyaeHbnfgDOvwBHrxAJfwnaebbnrfifHhDYfgasaacH8akY=wiFfYdH8Gipec8Eeeu0xXdbba9frFj0=OqFfea0dXdd9vqai=hGuQ8kuc9pgc9s8qqaq=dirpe0xb9q8qiLsFr0=vr0=vr0dc8meaabaqaciaacaGaaeqabaWaaeGaeaaakeaaimaacqWFWesraaa@3785@, *f*), where V
 MathType@MTEF@5@5@+=feaafiart1ev1aaatCvAUfKttLearuWrP9MDH5MBPbIqV92AaeXatLxBI9gBamrtHrhAL1wy0L2yHvtyaeHbnfgDOvwBHrxAJfwnaebbnrfifHhDYfgasaacH8akY=wiFfYdH8Gipec8Eeeu0xXdbba9frFj0=OqFfea0dXdd9vqai=hGuQ8kuc9pgc9s8qqaq=dirpe0xb9q8qiLsFr0=vr0=vr0dc8meaabaqaciaacaGaaeqabaWaaeGaeaaakeaaimaacqWFveVvaaa@384B@ is a vertex set and ℰ
 MathType@MTEF@5@5@+=feaafiart1ev1aaatCvAUfKttLearuWrP9MDH5MBPbIqV92AaeXatLxBI9gBamrtHrhAL1wy0L2yHvtyaeHbnfgDOvwBHrxAJfwnaebbnrfifHhDYfgasaacH8akY=wiFfYdH8Gipec8Eeeu0xXdbba9frFj0=OqFfea0dXdd9vqai=hGuQ8kuc9pgc9s8qqaq=dirpe0xb9q8qiLsFr0=vr0=vr0dc8meaabaqaciaacaGaaeqabaWaaeGaeaaakeaaimaacqWFWesraaa@3785@ is an edge set. *f *is a vertex-labeling function *f*: V
 MathType@MTEF@5@5@+=feaafiart1ev1aaatCvAUfKttLearuWrP9MDH5MBPbIqV92AaeXatLxBI9gBamrtHrhAL1wy0L2yHvtyaeHbnfgDOvwBHrxAJfwnaebbnrfifHhDYfgasaacH8akY=wiFfYdH8Gipec8Eeeu0xXdbba9frFj0=OqFfea0dXdd9vqai=hGuQ8kuc9pgc9s8qqaq=dirpe0xb9q8qiLsFr0=vr0=vr0dc8meaabaqaciaacaGaaeqabaWaaeGaeaaakeaaimaacqWFveVvaaa@384B@ → ℒ
 MathType@MTEF@5@5@+=feaafiart1ev1aaatCvAUfKttLearuWrP9MDH5MBPbIqV92AaeXatLxBI9gBamrtHrhAL1wy0L2yHvtyaeHbnfgDOvwBHrxAJfwnaebbnrfifHhDYfgasaacH8akY=wiFfYdH8Gipec8Eeeu0xXdbba9frFj0=OqFfea0dXdd9vqai=hGuQ8kuc9pgc9s8qqaq=dirpe0xb9q8qiLsFr0=vr0=vr0dc8meaabaqaciaacaGaaeqabaWaaeGaeaaakeaaimaacqWFsectaaa@376E@, where ℒ
 MathType@MTEF@5@5@+=feaafiart1ev1aaatCvAUfKttLearuWrP9MDH5MBPbIqV92AaeXatLxBI9gBamrtHrhAL1wy0L2yHvtyaeHbnfgDOvwBHrxAJfwnaebbnrfifHhDYfgasaacH8akY=wiFfYdH8Gipec8Eeeu0xXdbba9frFj0=OqFfea0dXdd9vqai=hGuQ8kuc9pgc9s8qqaq=dirpe0xb9q8qiLsFr0=vr0=vr0dc8meaabaqaciaacaGaaeqabaWaaeGaeaaakeaaimaacqWFsectaaa@376E@ = {ℓ_*l*_} is a set of possible labels for vertices.

For an organism *O*_*i*_, each vertex *v *∈ V
 MathType@MTEF@5@5@+=feaafiart1ev1aaatCvAUfKttLearuWrP9MDH5MBPbIqV92AaeXatLxBI9gBamrtHrhAL1wy0L2yHvtyaeHbnfgDOvwBHrxAJfwnaebbnrfifHhDYfgasaacH8akY=wiFfYdH8Gipec8Eeeu0xXdbba9frFj0=OqFfea0dXdd9vqai=hGuQ8kuc9pgc9s8qqaq=dirpe0xb9q8qiLsFr0=vr0=vr0dc8meaabaqaciaacaGaaeqabaWaaeGaeaaakeaaimaacqWFveVvaaa@384B@_*i *_corresponds to an enzyme of *O*_*i*_, and the cardinality |V
 MathType@MTEF@5@5@+=feaafiart1ev1aaatCvAUfKttLearuWrP9MDH5MBPbIqV92AaeXatLxBI9gBamrtHrhAL1wy0L2yHvtyaeHbnfgDOvwBHrxAJfwnaebbnrfifHhDYfgasaacH8akY=wiFfYdH8Gipec8Eeeu0xXdbba9frFj0=OqFfea0dXdd9vqai=hGuQ8kuc9pgc9s8qqaq=dirpe0xb9q8qiLsFr0=vr0=vr0dc8meaabaqaciaacaGaaeqabaWaaeGaeaaakeaaimaacqWFveVvaaa@384B@_*i*_| is equal to the number of distinct enzymes in the enzyme-enzyme relation list *R*(*O*_*i*_) of the organism. When an entry for two enzymes *e*_*u *_and *e*_*v *_is found in *R*(*O*_*i*_), the corresponding vertices *u *and *v *are directly connected by an edge (*u*, *v*) ∈ ℰ
 MathType@MTEF@5@5@+=feaafiart1ev1aaatCvAUfKttLearuWrP9MDH5MBPbIqV92AaeXatLxBI9gBamrtHrhAL1wy0L2yHvtyaeHbnfgDOvwBHrxAJfwnaebbnrfifHhDYfgasaacH8akY=wiFfYdH8Gipec8Eeeu0xXdbba9frFj0=OqFfea0dXdd9vqai=hGuQ8kuc9pgc9s8qqaq=dirpe0xb9q8qiLsFr0=vr0=vr0dc8meaabaqaciaacaGaaeqabaWaaeGaeaaakeaaimaacqWFWesraaa@3785@_*i *_(denoted by *u *~ *v*). The set ℒ
 MathType@MTEF@5@5@+=feaafiart1ev1aaatCvAUfKttLearuWrP9MDH5MBPbIqV92AaeXatLxBI9gBamrtHrhAL1wy0L2yHvtyaeHbnfgDOvwBHrxAJfwnaebbnrfifHhDYfgasaacH8akY=wiFfYdH8Gipec8Eeeu0xXdbba9frFj0=OqFfea0dXdd9vqai=hGuQ8kuc9pgc9s8qqaq=dirpe0xb9q8qiLsFr0=vr0=vr0dc8meaabaqaciaacaGaaeqabaWaaeGaeaaakeaaimaacqWFsectaaa@376E@ contains the unique identifiers (i.e., EC numbers) of all enzymes found in {R(Oi)}i=1N
 MathType@MTEF@5@5@+=feaafiart1ev1aaatCvAUfKttLearuWrP9MDH5MBPbIqV92AaeXatLxBI9gBaebbnrfifHhDYfgasaacH8akY=wiFfYdH8Gipec8Eeeu0xXdbba9frFj0=OqFfea0dXdd9vqai=hGuQ8kuc9pgc9s8qqaq=dirpe0xb9q8qiLsFr0=vr0=vr0dc8meaabaqaciaacaGaaeqabaqabeGadaaakeaadaGadaqaaiabdkfasnaabmaabaGaem4ta80aaSbaaSqaaiabdMgaPbqabaaakiaawIcacaGLPaaaaiaawUhacaGL9baadaqhaaWcbaGaemyAaKMaeyypa0JaeGymaedabaGaemOta4eaaaaa@38EE@ of all selected organisms.

A matrix representation of a labeled graph Γ_*i *_can be given by an adjacency matrix *H*_*i *_and a label matrix *L*_*i *_and, where *H*_*i *_is a |V
 MathType@MTEF@5@5@+=feaafiart1ev1aaatCvAUfKttLearuWrP9MDH5MBPbIqV92AaeXatLxBI9gBamrtHrhAL1wy0L2yHvtyaeHbnfgDOvwBHrxAJfwnaebbnrfifHhDYfgasaacH8akY=wiFfYdH8Gipec8Eeeu0xXdbba9frFj0=OqFfea0dXdd9vqai=hGuQ8kuc9pgc9s8qqaq=dirpe0xb9q8qiLsFr0=vr0=vr0dc8meaabaqaciaacaGaaeqabaWaaeGaeaaakeaaimaacqWFveVvaaa@384B@_*i*_| × |V
 MathType@MTEF@5@5@+=feaafiart1ev1aaatCvAUfKttLearuWrP9MDH5MBPbIqV92AaeXatLxBI9gBamrtHrhAL1wy0L2yHvtyaeHbnfgDOvwBHrxAJfwnaebbnrfifHhDYfgasaacH8akY=wiFfYdH8Gipec8Eeeu0xXdbba9frFj0=OqFfea0dXdd9vqai=hGuQ8kuc9pgc9s8qqaq=dirpe0xb9q8qiLsFr0=vr0=vr0dc8meaabaqaciaacaGaaeqabaWaaeGaeaaakeaaimaacqWFveVvaaa@384B@_*i*_| square matrix and *L*_*i *_is a |ℒ
 MathType@MTEF@5@5@+=feaafiart1ev1aaatCvAUfKttLearuWrP9MDH5MBPbIqV92AaeXatLxBI9gBamrtHrhAL1wy0L2yHvtyaeHbnfgDOvwBHrxAJfwnaebbnrfifHhDYfgasaacH8akY=wiFfYdH8Gipec8Eeeu0xXdbba9frFj0=OqFfea0dXdd9vqai=hGuQ8kuc9pgc9s8qqaq=dirpe0xb9q8qiLsFr0=vr0=vr0dc8meaabaqaciaacaGaaeqabaWaaeGaeaaakeaaimaacqWFsectaaa@376E@| × |V
 MathType@MTEF@5@5@+=feaafiart1ev1aaatCvAUfKttLearuWrP9MDH5MBPbIqV92AaeXatLxBI9gBamrtHrhAL1wy0L2yHvtyaeHbnfgDOvwBHrxAJfwnaebbnrfifHhDYfgasaacH8akY=wiFfYdH8Gipec8Eeeu0xXdbba9frFj0=OqFfea0dXdd9vqai=hGuQ8kuc9pgc9s8qqaq=dirpe0xb9q8qiLsFr0=vr0=vr0dc8meaabaqaciaacaGaaeqabaWaaeGaeaaakeaaimaacqWFveVvaaa@384B@_*i*_| matrix. Each element *H*_*i*_(*a*, *b*) is given by

Hi(a,b)={w(va,vb)if va∼vb0otherwise     (1)
 MathType@MTEF@5@5@+=feaafiart1ev1aaatCvAUfKttLearuWrP9MDH5MBPbIqV92AaeXatLxBI9gBaebbnrfifHhDYfgasaacH8akY=wiFfYdH8Gipec8Eeeu0xXdbba9frFj0=OqFfea0dXdd9vqai=hGuQ8kuc9pgc9s8qqaq=dirpe0xb9q8qiLsFr0=vr0=vr0dc8meaabaqaciaacaGaaeqabaqabeGadaaakeaacqWGibasdaWgaaWcbaGaemyAaKgabeaakmaabmaabaGaemyyaeMaeiilaWIaemOyaigacaGLOaGaayzkaaGaeyypa0ZaaiqaaeaafaqabeGacaaabaGaem4DaC3aaSbaaSqaaiabcIcaOiabdAha2naaBaaameaacqWGHbqyaeqaaSGaeiilaWIaemODay3aaSbaaWqaaiabdkgaIbqabaWccqGGPaqkaeqaaaGcbaGaeeyAaKMaeeOzayMaeeiiaaIaemODay3aaSbaaSqaaiabdggaHbqabaGccqWI8iIocqWG2bGDdaWgaaWcbaGaemOyaigabeaaaOqaaiabicdaWaqaaiabb+gaVjabbsha0jabbIgaOjabbwgaLjabbkhaYjabbEha3jabbMgaPjabbohaZjabbwgaLbaaaiaawUhaaiaaxMaacaWLjaWaaeWaaeaacqaIXaqmaiaawIcacaGLPaaaaaa@5C60@

where w(va,vb)
 MathType@MTEF@5@5@+=feaafiart1ev1aaatCvAUfKttLearuWrP9MDH5MBPbIqV92AaeXatLxBI9gBaebbnrfifHhDYfgasaacH8akY=wiFfYdH8Gipec8Eeeu0xXdbba9frFj0=OqFfea0dXdd9vqai=hGuQ8kuc9pgc9s8qqaq=dirpe0xb9q8qiLsFr0=vr0=vr0dc8meaabaqaciaacaGaaeqabaqabeGadaaakeaacqWG3bWDdaWgaaWcbaGaeiikaGIaemODay3aaSbaaWqaaiabdggaHbqabaWccqGGSaalcqWG2bGDdaWgaaadbaGaemOyaigabeaaliabcMcaPaqabaaaaa@36D3@ is the weight of the edge (*v*_*a*_, *v*_*b*_). Whenever the vertices *v*_*a *_and *v*_*b *_are joined by an edge, we set the weight such that *w*(*v*_*a*_, *v*_*b*_) = CΓi⋅1deg(va)
 MathType@MTEF@5@5@+=feaafiart1ev1aaatCvAUfKttLearuWrP9MDH5MBPbIqV92AaeXatLxBI9gBaebbnrfifHhDYfgasaacH8akY=wiFfYdH8Gipec8Eeeu0xXdbba9frFj0=OqFfea0dXdd9vqai=hGuQ8kuc9pgc9s8qqaq=dirpe0xb9q8qiLsFr0=vr0=vr0dc8meaabaqaciaacaGaaeqabaqabeGadaaakeaacqWGdbWqdaWgaaWcbaGaeu4KdC0aaSbaaWqaaiabdMgaPbqabaaaleqaaOGaeyyXIC9aaSaaaeaacqaIXaqmaeaaieGacqWFKbazcqWFLbqzcqWFNbWzcqGGOaakcqWG2bGDdaWgaaWcbaGaemyyaegabeaakiabcMcaPaaaaaa@3CD8@, where *deg*(*v*_*a*_) is the degree of *v*_*a *_and CΓi
 MathType@MTEF@5@5@+=feaafiart1ev1aaatCvAUfKttLearuWrP9MDH5MBPbIqV92AaeXatLxBI9gBaebbnrfifHhDYfgasaacH8akY=wiFfYdH8Gipec8Eeeu0xXdbba9frFj0=OqFfea0dXdd9vqai=hGuQ8kuc9pgc9s8qqaq=dirpe0xb9q8qiLsFr0=vr0=vr0dc8meaabaqaciaacaGaaeqabaqabeGadaaakeaacqWGdbWqdaWgaaWcbaGaeu4KdC0aaSbaaWqaaiabdMgaPbqabaaaleqaaaaa@30E2@ is a constant for the graph Γ_*i*_. Then, *w*(*v*_*a*_, *v*_*b*_) can be thought to be proportional to a probability (1/*deg*(*v*_*a*_)) to visit *v*_*b *_in one step in a random walk starting from *v*_*a*_. We set CΓi=∑v∈Videg(v)|Vi|
 MathType@MTEF@5@5@+=feaafiart1ev1aaatCvAUfKttLearuWrP9MDH5MBPbIqV92AaeXatLxBI9gBamrtHrhAL1wy0L2yHvtyaeHbnfgDOvwBHrxAJfwnaebbnrfifHhDYfgasaacH8akY=wiFfYdH8Gipec8Eeeu0xXdbba9frFj0=OqFfea0dXdd9vqai=hGuQ8kuc9pgc9s8qqaq=dirpe0xb9q8qiLsFr0=vr0=vr0dc8meaabaqaciaacaGaaeqabaWaaeGaeaaakeaacqWGdbWqdaWgaaWcbaGaeu4KdC0aaSbaaWqaaiabdMgaPbqabaaaleqaaOGaeyypa0ZaaSaaaeaadaaeqaqaaGqaciab=rgaKjab=vgaLjab=DgaNjabcIcaOiabdAha2jabcMcaPaWcbaGaemODayNaeyicI4mcdaGae4xfXB1aaSbaaWqaaiab=LgaPbqabaaaleqaniabggHiLdaakeaacqGG8baFcqGFveVvdaWgaaWcbaGae8xAaKgabeaakiabcYha8baaaaa@51A3@, which makes *H*_*i *_such that its total sum of elemets is still same to the number of edges in a bidirectional representation of Γ_*i*_.

An element of the matrix *L*_*i *_defined as

Li(l,a)={1if ℓl=f(va),0otherwise.     (2)
 MathType@MTEF@5@5@+=feaafiart1ev1aaatCvAUfKttLearuWrP9MDH5MBPbIqV92AaeXatLxBI9gBaebbnrfifHhDYfgasaacH8akY=wiFfYdH8Gipec8Eeeu0xXdbba9frFj0=OqFfea0dXdd9vqai=hGuQ8kuc9pgc9s8qqaq=dirpe0xb9q8qiLsFr0=vr0=vr0dc8meaabaqaciaacaGaaeqabaqabeGadaaakeaacqWGmbatdaWgaaWcbaGaemyAaKgabeaakiabcIcaOiabdYgaSjabcYcaSiabdggaHjabcMcaPiabg2da9maaceaabaqbaeaabiGaaaqaaiabigdaXaqaaiabbMgaPjabbAgaMjabbccaGiabloriSnaaBaaaleaacqWGSbaBaeqaaOGaeyypa0JaemOzayMaeiikaGIaemODay3aaSbaaSqaaiabdggaHbqabaGccqGGPaqkcqGGSaalaeaacqaIWaamaeaacqqGVbWBcqqG0baDcqqGObaAcqqGLbqzcqqGYbGCcqqG3bWDcqqGPbqAcqqGZbWCcqqGLbqzcqGGUaGlaaaacaGL7baacaWLjaGaaCzcamaabmaabaGaeGOmaidacaGLOaGaayzkaaaaaa@57DD@

with 1 ≤ *l *≤ |ℒ
 MathType@MTEF@5@5@+=feaafiart1ev1aaatCvAUfKttLearuWrP9MDH5MBPbIqV92AaeXatLxBI9gBamrtHrhAL1wy0L2yHvtyaeHbnfgDOvwBHrxAJfwnaebbnrfifHhDYfgasaacH8akY=wiFfYdH8Gipec8Eeeu0xXdbba9frFj0=OqFfea0dXdd9vqai=hGuQ8kuc9pgc9s8qqaq=dirpe0xb9q8qiLsFr0=vr0=vr0dc8meaabaqaciaacaGaaeqabaWaaeGaeaaakeaaimaacqWFsectaaa@376E@|, 1 ≤ *a *≤ |V
 MathType@MTEF@5@5@+=feaafiart1ev1aaatCvAUfKttLearuWrP9MDH5MBPbIqV92AaeXatLxBI9gBamrtHrhAL1wy0L2yHvtyaeHbnfgDOvwBHrxAJfwnaebbnrfifHhDYfgasaacH8akY=wiFfYdH8Gipec8Eeeu0xXdbba9frFj0=OqFfea0dXdd9vqai=hGuQ8kuc9pgc9s8qqaq=dirpe0xb9q8qiLsFr0=vr0=vr0dc8meaabaqaciaacaGaaeqabaWaaeGaeaaakeaaimaacqWFveVvaaa@384B@_*i*_|. This means that *L*_*i*_(*l*, *a*) is 1 only when the label of vertex *v*_*a *_is ℓ_*l*_. Since we represent a metabolic pathway in such a way that every vertex (enzyme) in it has a unique EC number, every column sum of *L*_*i *_is 1, that is, ∑_*l*_*L*_*i*_(*l*, *a*) = 1, (∀ *a*). And, in terms of rows of *L*, ∑_*a*_*L*_*i*_(*l*, *a*) = 1 if ℓ_*l *_= *f*(*v*) (∃ *v *∈ V
 MathType@MTEF@5@5@+=feaafiart1ev1aaatCvAUfKttLearuWrP9MDH5MBPbIqV92AaeXatLxBI9gBamrtHrhAL1wy0L2yHvtyaeHbnfgDOvwBHrxAJfwnaebbnrfifHhDYfgasaacH8akY=wiFfYdH8Gipec8Eeeu0xXdbba9frFj0=OqFfea0dXdd9vqai=hGuQ8kuc9pgc9s8qqaq=dirpe0xb9q8qiLsFr0=vr0=vr0dc8meaabaqaciaacaGaaeqabaWaaeGaeaaakeaaimaacqWFveVvaaa@384B@_*i*_); ∑_*a*_*L*_*i*_(*l*, *a*) = 0 otherwise. To compare the structures between metabolic networks of two organisms represented in graphs as described above, we adopted a kernel-based approach called the *exponential graph kernel *[39].

#### Comparison of metabolic networks: graph kernel

Given two graphs Γ_*i *_= (*L*_*i*_, *H*_*i*_) and Γ_*j *_= (*L*_*j*_, *H*_*j*_), the first simple approach to the graph comparison is to count the common vertices with the same labels in both Γ_*i *_and Γ_*j*_. This similarity (or kernel) can be calculated by *k*(Γ_*i*_, Γ_*j*_) = <LiLiT
 MathType@MTEF@5@5@+=feaafiart1ev1aaatCvAUfKttLearuWrP9MDH5MBPbIqV92AaeXatLxBI9gBaebbnrfifHhDYfgasaacH8akY=wiFfYdH8Gipec8Eeeu0xXdbba9frFj0=OqFfea0dXdd9vqai=hGuQ8kuc9pgc9s8qqaq=dirpe0xb9q8qiLsFr0=vr0=vr0dc8meaabaqaciaacaGaaeqabaqabeGadaaakeaacqWGmbatdaWgaaWcbaGaemyAaKgabeaakiabdYeamnaaDaaaleaacqWGPbqAaeaacqWGubavaaaaaa@3338@, LjLjT
 MathType@MTEF@5@5@+=feaafiart1ev1aaatCvAUfKttLearuWrP9MDH5MBPbIqV92AaeXatLxBI9gBaebbnrfifHhDYfgasaacH8akY=wiFfYdH8Gipec8Eeeu0xXdbba9frFj0=OqFfea0dXdd9vqai=hGuQ8kuc9pgc9s8qqaq=dirpe0xb9q8qiLsFr0=vr0=vr0dc8meaabaqaciaacaGaaeqabaqabeGadaaakeaacqWGmbatdaWgaaWcbaGaemOAaOgabeaakiabdYeamnaaDaaaleaacqWGQbGAaeaacqWGubavaaaaaa@333C@>, where the inner product <*M*_*i*_, *M*_*j*_> between two matrices of the same dimension is defined as

〈Mi,Mj〉=∑a∑bMi(a,b)×Mj(a,b).     (3)
 MathType@MTEF@5@5@+=feaafiart1ev1aaatCvAUfKttLearuWrP9MDH5MBPbIqV92AaeXatLxBI9gBaebbnrfifHhDYfgasaacH8akY=wiFfYdH8Gipec8Eeeu0xXdbba9frFj0=OqFfea0dXdd9vqai=hGuQ8kuc9pgc9s8qqaq=dirpe0xb9q8qiLsFr0=vr0=vr0dc8meaabaqaciaacaGaaeqabaqabeGadaaakeaacqGHPms4cqWGnbqtdaWgaaWcbaGaemyAaKgabeaakiabcYcaSiabd2eannaaBaaaleaacqWGQbGAaeqaaOGaeyOkJeVaeyypa0Zaaabuaeaadaaeqbqaaiabd2eannaaBaaaleaacqWGPbqAaeqaaOGaeiikaGIaemyyaeMaeiilaWIaemOyaiMaeiykaKIaey41aqRaemyta00aaSbaaSqaaiabdQgaQbqabaGccqGGOaakcqWGHbqycqGGSaalcqWGIbGycqGGPaqkaSqaaiabdkgaIbqab0GaeyyeIuoaaSqaaiabdggaHbqab0GaeyyeIuoakiabc6caUiaaxMaacaWLjaWaaeWaaeaacqaIZaWmaiaawIcacaGLPaaaaaa@54E1@

Based on the definition of the label matrix in Equation (2), the matrix *M*_*i *_= LiLiT
 MathType@MTEF@5@5@+=feaafiart1ev1aaatCvAUfKttLearuWrP9MDH5MBPbIqV92AaeXatLxBI9gBaebbnrfifHhDYfgasaacH8akY=wiFfYdH8Gipec8Eeeu0xXdbba9frFj0=OqFfea0dXdd9vqai=hGuQ8kuc9pgc9s8qqaq=dirpe0xb9q8qiLsFr0=vr0=vr0dc8meaabaqaciaacaGaaeqabaqabeGadaaakeaacqWGmbatdaWgaaWcbaGaemyAaKgabeaakiabdYeamnaaDaaaleaacqWGPbqAaeaacqWGubavaaaaaa@3338@ is a |ℒ
 MathType@MTEF@5@5@+=feaafiart1ev1aaatCvAUfKttLearuWrP9MDH5MBPbIqV92AaeXatLxBI9gBamrtHrhAL1wy0L2yHvtyaeHbnfgDOvwBHrxAJfwnaebbnrfifHhDYfgasaacH8akY=wiFfYdH8Gipec8Eeeu0xXdbba9frFj0=OqFfea0dXdd9vqai=hGuQ8kuc9pgc9s8qqaq=dirpe0xb9q8qiLsFr0=vr0=vr0dc8meaabaqaciaacaGaaeqabaWaaeGaeaaakeaaimaacqWFsectaaa@376E@| × |ℒ
 MathType@MTEF@5@5@+=feaafiart1ev1aaatCvAUfKttLearuWrP9MDH5MBPbIqV92AaeXatLxBI9gBamrtHrhAL1wy0L2yHvtyaeHbnfgDOvwBHrxAJfwnaebbnrfifHhDYfgasaacH8akY=wiFfYdH8Gipec8Eeeu0xXdbba9frFj0=OqFfea0dXdd9vqai=hGuQ8kuc9pgc9s8qqaq=dirpe0xb9q8qiLsFr0=vr0=vr0dc8meaabaqaciaacaGaaeqabaWaaeGaeaaakeaaimaacqWFsectaaa@376E@| diagonal matrix where *M*_*i*_(*l*, *l*) = 1 only when *f*(*v*) = ℓ_*l *_(∃*v *∈ V
 MathType@MTEF@5@5@+=feaafiart1ev1aaatCvAUfKttLearuWrP9MDH5MBPbIqV92AaeXatLxBI9gBamrtHrhAL1wy0L2yHvtyaeHbnfgDOvwBHrxAJfwnaebbnrfifHhDYfgasaacH8akY=wiFfYdH8Gipec8Eeeu0xXdbba9frFj0=OqFfea0dXdd9vqai=hGuQ8kuc9pgc9s8qqaq=dirpe0xb9q8qiLsFr0=vr0=vr0dc8meaabaqaciaacaGaaeqabaWaaeGaeaaakeaaimaacqWFveVvaaa@384B@_*i*_), and *M*_*i*_(*l*, *l*) = 0 otherwise. However, this approach considers only the presence or absence of vertices (enzymes) but does not consider the structure of the graph, such that the successive enzymes or reaction steps cannot be considered when comparing metabolic networks. To capture the structure of the graph, one must also consider vertices that can be reached from a vertex by a subsequent traverse.

In the exponential graph-kernel method, the similarity between two graphs Γ_*i *_and Γ_*j *_is defined as

k(Γi,Γj)=〈LieβHiLiT,LjeβHjLjT〉,     (4)eβH=∑n=0∞(βH)nn!=I+βH+β22!H2+⋯,     (5)
 MathType@MTEF@5@5@+=feaafiart1ev1aaatCvAUfKttLearuWrP9MDH5MBPbIqV92AaeXatLxBI9gBaebbnrfifHhDYfgasaacH8akY=wiFfYdH8Gipec8Eeeu0xXdbba9frFj0=OqFfea0dXdd9vqai=hGuQ8kuc9pgc9s8qqaq=dirpe0xb9q8qiLsFr0=vr0=vr0dc8meaabaqaciaacaGaaeqabaqabeGadaaakeGacea2jea=muaabaqadmaaaeaacqWGRbWAcqGGOaakcqqHtoWrdaWgaaWcbaGaemyAaKgabeaakiabcYcaSiabfo5ahnaaBaaaleaacqWGQbGAaeqaaOGaeiykaKcabaGaeyypa0dabaGaeyykJeUaemitaW0aaSbaaSqaaiabdMgaPbqabaGccqWGLbqzdaahaaWcbeqaaGGaciab=j7aIjabdIeainaaBaaameaacqWGPbqAaeqaaaaakiabdYeamnaaDaaaleaacqWGPbqAaeaacqWGubavaaGccqGGSaalcqWGmbatdaWgaaWcbaGaemOAaOgabeaakiabdwgaLnaaCaaaleqabaGae8NSdiMaemisaG0aaSbaaWqaaiabdQgaQbqabaaaaOGaemitaW0aa0baaSqaaiabdQgaQbqaaiabdsfaubaakiabgQYiXlabcYcaSiaaxMaacaWLjaWaaeWaaeaacqaI0aanaiaawIcacaGLPaaaaeGabea6ciaaxMaacqWGLbqzdaahaaWcbeqaaiab=j7aIjabdIeaibaaaOqaaiabg2da9aqacmqajJFavJpa5J=aaabCaeaadaWcaaqaaiabcIcaOiab=j7aIjabdIeaijabcMcaPmaaCaaaleqabaGaemOBa4gaaaGcbaGaemOBa4MaeiyiaecaaaWcbaGaemOBa4Maeyypa0JaeGimaadabaGaeyOhIukaniabggHiLdaakeaaaeaacqGH9aqpaeaacqWGjbqscqGHRaWkcqWFYoGycqWGibascqGHRaWkdaWcaaqaaiab=j7aInaaCaaaleqabaGaeGOmaidaaaGcbaGaeGOmaiJaeiyiaecaaiabdIeainaaCaaaleqabaGaeGOmaidaaOGaey4kaSIaeS47IWKaeiilaWIaaCzcaiaaxMaadaqadaqaceaaooVaeGynaudacaGLOaGaayzkaaaaaaaa@8D87@

where *β *(≥ 0) is a real-valued parameter and its value is chosen by performing many tries. When *β *= 0, it recovers the simple common vertex-counting measure since exp(0*H*) = *I*, the |V
 MathType@MTEF@5@5@+=feaafiart1ev1aaatCvAUfKttLearuWrP9MDH5MBPbIqV92AaeXatLxBI9gBamrtHrhAL1wy0L2yHvtyaeHbnfgDOvwBHrxAJfwnaebbnrfifHhDYfgasaacH8akY=wiFfYdH8Gipec8Eeeu0xXdbba9frFj0=OqFfea0dXdd9vqai=hGuQ8kuc9pgc9s8qqaq=dirpe0xb9q8qiLsFr0=vr0=vr0dc8meaabaqaciaacaGaaeqabaWaaeGaeaaakeaaimaacqWFveVvaaa@384B@| × |V
 MathType@MTEF@5@5@+=feaafiart1ev1aaatCvAUfKttLearuWrP9MDH5MBPbIqV92AaeXatLxBI9gBamrtHrhAL1wy0L2yHvtyaeHbnfgDOvwBHrxAJfwnaebbnrfifHhDYfgasaacH8akY=wiFfYdH8Gipec8Eeeu0xXdbba9frFj0=OqFfea0dXdd9vqai=hGuQ8kuc9pgc9s8qqaq=dirpe0xb9q8qiLsFr0=vr0=vr0dc8meaabaqaciaacaGaaeqabaWaaeGaeaaakeaaimaacqWFveVvaaa@384B@| identity matrix. Each element *H*^*n*^(*a*, *b*) of the matrix *H*^*n *^in Equation (5) represents the number of walks of length *n *(admitting cycles) from *v*_*a *_to *v*_*b*_, and allows the representation of the global structure of a graph.

Substituting Equation (5) into Equation (4), we can decompose the kernel function *k*(Γ_*i*_, Γ_*j*_) into two meaningful parts, *k*(Γ_*i*_, Γ_*j*_) = *k*_1_(Γ_*i*_, Γ_*j*_) + *k*_2_(Γ_*i*_, Γ_*j*_), where

k1=∑n=0∞βn+βnn!n!〈LiHinLiT,LjHjnLjT〉     (6)
 MathType@MTEF@5@5@+=feaafiart1ev1aaatCvAUfKttLearuWrP9MDH5MBPbIqV92AaeXatLxBI9gBaebbnrfifHhDYfgasaacH8akY=wiFfYdH8Gipec8Eeeu0xXdbba9frFj0=OqFfea0dXdd9vqai=hGuQ8kuc9pgc9s8qqaq=dirpe0xb9q8qiLsFr0=vr0=vr0dc8meaabaqaciaacaGaaeqabaqabeGadaaakeaacqWGRbWAdaWgaaWcbaGaeGymaedabeaakiabg2da9maaqahabaWaaSaaaeaacqaHYoGydaahaaWcbeqaaiabd6gaUbaakiabgUcaRiabek7aInaaCaaaleqabaGaemOBa4gaaaGcbaGaemOBa4MaeiyiaeIaemOBa4MaeiyiaecaaaWcbaGaemOBa4Maeyypa0JaeGimaadabaGaeyOhIukaniabggHiLdGccqGHPms4cqWGmbatdaWgaaWcbaGaemyAaKgabeaakiabdIeainaaDaaaleaacqWGPbqAaeaacqWGUbGBaaGccqWGmbatdaqhaaWcbaGaemyAaKgabaGaemivaqfaaOGaeiilaWIaemitaW0aaSbaaSqaaiabdQgaQbqabaGccqWGibasdaqhaaWcbaGaemOAaOgabaGaemOBa4gaaOGaemitaW0aa0baaSqaaiabdQgaQbqaaiabdsfaubaakiabgQYiXlaaxMaacaWLjaWaaeWaaeaacqaI2aGnaiaawIcacaGLPaaaaaa@6093@

k2=∑n=m=0,n≠m∞βnβmn!m!〈LiHinLiT,LjHjmLjT〉.     (7)
 MathType@MTEF@5@5@+=feaafiart1ev1aaatCvAUfKttLearuWrP9MDH5MBPbIqV92AaeXatLxBI9gBaebbnrfifHhDYfgasaacH8akY=wiFfYdH8Gipec8Eeeu0xXdbba9frFj0=OqFfea0dXdd9vqai=hGuQ8kuc9pgc9s8qqaq=dirpe0xb9q8qiLsFr0=vr0=vr0dc8meaabaqaciaacaGaaeqabaqabeGadaaakeaacqWGRbWAdaWgaaWcbaGaeGOmaidabeaakiabg2da9maaqahabaWaaSaaaeaaiiGacqWFYoGydaahaaWcbeqaaiabd6gaUbaakiab=j7aInaaCaaaleqabaGaemyBa0gaaaGcbaGaemOBa4MaeiyiaeIaemyBa0MaeiyiaecaaaWcbaGaemOBa4Maeyypa0JaemyBa0Maeyypa0JaeGimaaJaeiilaWIaemOBa4MaeyiyIKRaemyBa0gabaGaeyOhIukaniabggHiLdGccqGHPms4cqWGmbatdaWgaaWcbaGaemyAaKgabeaakiabdIeainaaDaaaleaacqWGPbqAaeaacqWGUbGBaaGccqWGmbatdaqhaaWcbaGaemyAaKgabaGaemivaqfaaOGaeiilaWIaemitaW0aaSbaaSqaaiabdQgaQbqabaGccqWGibasdaqhaaWcbaGaemOAaOgabaGaemyBa0gaaOGaemitaW0aa0baaSqaaiabdQgaQbqaaiabdsfaubaakiabgQYiXlabc6caUiaaxMaacaWLjaWaaeWaaeaacqaI3aWnaiaawIcacaGLPaaaaaa@686D@

The kernel function *k*_1 _contributes by considering walks of the same length in both graphs, and *k*_2 _can take into account the insertion or deletion of vertices in the graph [39]. As the number of movements in a graph increases, the significance of walks of length *n *decreases by βnn!
 MathType@MTEF@5@5@+=feaafiart1ev1aaatCvAUfKttLearuWrP9MDH5MBPbIqV92AaeXatLxBI9gBaebbnrfifHhDYfgasaacH8akY=wiFfYdH8Gipec8Eeeu0xXdbba9frFj0=OqFfea0dXdd9vqai=hGuQ8kuc9pgc9s8qqaq=dirpe0xb9q8qiLsFr0=vr0=vr0dc8meaabaqaciaacaGaaeqabaqabeGadaaakeaadaWcaaqaaGGaciab=j7aInaaCaaaleqabaGaemOBa4gaaaGcbaGaemOBa4Maeiyiaecaaaaa@322F@. Eventually, the exponential matrix *e*^*βH *^can be interpreted as the product of a continuous process *H*, from which the identity matrix expands gradually to the matrix of the global structure of Γ [40].

The exponential graph kernel requires the exponentiation of square matrices *H*s. This can be performed by matrix diagonalization, with time complexity of about *O*(|V
 MathType@MTEF@5@5@+=feaafiart1ev1aaatCvAUfKttLearuWrP9MDH5MBPbIqV92AaeXatLxBI9gBamrtHrhAL1wy0L2yHvtyaeHbnfgDOvwBHrxAJfwnaebbnrfifHhDYfgasaacH8akY=wiFfYdH8Gipec8Eeeu0xXdbba9frFj0=OqFfea0dXdd9vqai=hGuQ8kuc9pgc9s8qqaq=dirpe0xb9q8qiLsFr0=vr0=vr0dc8meaabaqaciaacaGaaeqabaWaaeGaeaaakeaaimaacqWFveVvaaa@384B@_*i*_|^3^) for *H*_*i *_thus Γ_*i*_. [39]. The time complexity of the element-wise product of two matrices in *k*(Γ_*i*_, Γ_*j*_) is *O*(max (|V
 MathType@MTEF@5@5@+=feaafiart1ev1aaatCvAUfKttLearuWrP9MDH5MBPbIqV92AaeXatLxBI9gBamrtHrhAL1wy0L2yHvtyaeHbnfgDOvwBHrxAJfwnaebbnrfifHhDYfgasaacH8akY=wiFfYdH8Gipec8Eeeu0xXdbba9frFj0=OqFfea0dXdd9vqai=hGuQ8kuc9pgc9s8qqaq=dirpe0xb9q8qiLsFr0=vr0=vr0dc8meaabaqaciaacaGaaeqabaWaaeGaeaaakeaaimaacqWFveVvaaa@384B@_*i*_|^2^, |V
 MathType@MTEF@5@5@+=feaafiart1ev1aaatCvAUfKttLearuWrP9MDH5MBPbIqV92AaeXatLxBI9gBamrtHrhAL1wy0L2yHvtyaeHbnfgDOvwBHrxAJfwnaebbnrfifHhDYfgasaacH8akY=wiFfYdH8Gipec8Eeeu0xXdbba9frFj0=OqFfea0dXdd9vqai=hGuQ8kuc9pgc9s8qqaq=dirpe0xb9q8qiLsFr0=vr0=vr0dc8meaabaqaciaacaGaaeqabaWaaeGaeaaakeaaimaacqWFveVvaaa@384B@_*j*_|^2^)). With *N *graphs, finally, the total time complexity for constructing the kernel matrix *K *= {*k*_*ij*_} (1 ≤ *i, j *≤ *N*) is *O*(*NV*^3 ^+ *N*^2^*V*^2^) where *V *= max_*i *_|V
 MathType@MTEF@5@5@+=feaafiart1ev1aaatCvAUfKttLearuWrP9MDH5MBPbIqV92AaeXatLxBI9gBamrtHrhAL1wy0L2yHvtyaeHbnfgDOvwBHrxAJfwnaebbnrfifHhDYfgasaacH8akY=wiFfYdH8Gipec8Eeeu0xXdbba9frFj0=OqFfea0dXdd9vqai=hGuQ8kuc9pgc9s8qqaq=dirpe0xb9q8qiLsFr0=vr0=vr0dc8meaabaqaciaacaGaaeqabaWaaeGaeaaakeaaimaacqWFveVvaaa@384B@_*i*_|.

From the kernel *k*(Γ_*i*_, Γ_*j*_), the dissimilarity metric is defined in the standard manner, that is,

d(Γi,Γj)=k(Γi,Γi)+k(Γj,Γj)−2k(Γi,Γj).     (8)
 MathType@MTEF@5@5@+=feaafiart1ev1aaatCvAUfKttLearuWrP9MDH5MBPbIqV92AaeXatLxBI9gBaebbnrfifHhDYfgasaacH8akY=wiFfYdH8Gipec8Eeeu0xXdbba9frFj0=OqFfea0dXdd9vqai=hGuQ8kuc9pgc9s8qqaq=dirpe0xb9q8qiLsFr0=vr0=vr0dc8meaabaqaciaacaGaaeqabaqabeGadaaakeaacqWGKbazcqGGOaakcqqHtoWrdaWgaaWcbaGaemyAaKgabeaakiabcYcaSiabfo5ahnaaBaaaleaacqWGQbGAaeqaaOGaeiykaKIaeyypa0ZaaOaaaeaacqWGRbWAcqGGOaakcqqHtoWrdaWgaaWcbaGaemyAaKgabeaakiabcYcaSiabfo5ahnaaBaaaleaacqWGPbqAaeqaaOGaeiykaKIaey4kaSIaem4AaSMaeiikaGIaeu4KdC0aaSbaaSqaaiabdQgaQbqabaGccqGGSaalcqqHtoWrdaWgaaWcbaGaemOAaOgabeaakiabcMcaPiabgkHiTiabikdaYiabdUgaRjabcIcaOiabfo5ahnaaBaaaleaacqWGPbqAaeqaaOGaeiilaWIaeu4KdC0aaSbaaSqaaiabdQgaQbqabaGccqGGPaqkaSqabaGccqGGUaGlcaWLjaGaaCzcamaabmaabaGaeGioaGdacaGLOaGaayzkaaaaaa@5CCD@

If we use the normalized kernel,

knorm(Γi,Γj)=k(Γi,Γj)k(Γi,Γi)k(Γj,Γj),     (9)
 MathType@MTEF@5@5@+=feaafiart1ev1aaatCvAUfKttLearuWrP9MDH5MBPbIqV92AaeXatLxBI9gBaebbnrfifHhDYfgasaacH8akY=wiFfYdH8Gipec8Eeeu0xXdbba9frFj0=OqFfea0dXdd9vqai=hGuQ8kuc9pgc9s8qqaq=dirpe0xb9q8qiLsFr0=vr0=vr0dc8meaabaqaciaacaGaaeqabaqabeGadaaakeaacqWGRbWAdaWgaaWcbaGaemOBa4Maem4Ba8MaemOCaiNaemyBa0gabeaakiabcIcaOiabfo5ahnaaBaaaleaacqWGPbqAaeqaaOGaeiilaWIaeu4KdC0aaSbaaSqaaiabdQgaQbqabaGccqGGPaqkcqGH9aqpdaWcaaqaaiabdUgaRjabcIcaOiabfo5ahnaaBaaaleaacqWGPbqAaeqaaOGaeiilaWIaeu4KdC0aaSbaaSqaaiabdQgaQbqabaGccqGGPaqkaeaadaGcaaqaaiabdUgaRjabcIcaOiabfo5ahnaaBaaaleaacqWGPbqAaeqaaOGaeiilaWIaeu4KdC0aaSbaaSqaaiabdMgaPbqabaGccqGGPaqkcqWGRbWAcqGGOaakcqqHtoWrdaWgaaWcbaGaemOAaOgabeaakiabcYcaSiabfo5ahnaaBaaaleaacqWGQbGAaeqaaOGaeiykaKcaleqaaaaakiabcYcaSiaaxMaacaWLjaWaaeWaaeaacqaI5aqoaiaawIcacaGLPaaaaaa@5FFA@

then the distance metric is simplified as

d(Γi,Γj)=2−2knorm(Γi,Γj).     (10)
 MathType@MTEF@5@5@+=feaafiart1ev1aaatCvAUfKttLearuWrP9MDH5MBPbIqV92AaeXatLxBI9gBaebbnrfifHhDYfgasaacH8akY=wiFfYdH8Gipec8Eeeu0xXdbba9frFj0=OqFfea0dXdd9vqai=hGuQ8kuc9pgc9s8qqaq=dirpe0xb9q8qiLsFr0=vr0=vr0dc8meaabaqaciaacaGaaeqabaqabeGadaaakeaacqWGKbazcqGGOaakcqqHtoWrdaWgaaWcbaGaemyAaKgabeaakiabcYcaSiabfo5ahnaaBaaaleaacqWGQbGAaeqaaOGaeiykaKIaeyypa0ZaaOaaaeaacqaIYaGmcqGHsislcqaIYaGmcqWGRbWAdaWgaaWcbaGaemOBa4Maem4Ba8MaemOCaiNaemyBa0gabeaakiabcIcaOiabfo5ahnaaBaaaleaacqWGPbqAaeqaaOGaeiilaWIaeu4KdC0aaSbaaSqaaiabdQgaQbqabaGccqGGPaqkaSqabaGccqGGUaGlcaWLjaGaaCzcamaabmaabaGaeGymaeJaeGimaadacaGLOaGaayzkaaaaaa@4FC5@

To summarize, metabolic networks constructed from reference pathways of *N *organisms were first converted to labeled undirected graphs. Each graph Γ_*i *_(1 ≤ *i *≤ *N*) was then represented by two matrices: the vertex-label matrix *L*_*i *_and the adjacency matrix *H*_*i*_. Using these two matrices, we can take into account only the local structure (the direct connectivities between enzymes in pathways) of networks. To compare networks in terms of their global structure, we adopted a kernel-based method, which we named the exponential graph kernel. Finally, the distance matrix acquired from the kernel function was fed into a hierarchical clustering algorithm to construct the phylogenetic trees.

#### Constructing phylogenetic trees

The distance between two organisms was calculated by comparing their metabolic networks using the measures mentioned earlier. To construct a phylogenetic tree, we used an unweighted pair-group method with arithmetic mean (UPGMA) [41,42], a hierarchical agglomerative clustering algorithm. Given *N *organisms, the algorithm starts by initializing *N *clusters, each of which contains exactly one distinct organism, and proceeds by iteratively merging the two nearest clusters until only one cluster (called the *root *of the tree) remains. The dendrogram derived from UPGMA is a binary tree, which we consider may represent a binary phylogenetic tree.

## Authors' contributions

SJO proposed the idea, organized overall procedure, built the data set for computational experiments and carried out an analysis of experimental results. JGJ built the data set for computational experiments and carried out an analysis of experimental results. JHC carried out implementation of the computational method on graph kernels, computational experiments and analysis. BTZ developed the idea, provided intellectual guidance and mentorship. All authors read and approved the final manuscript.

## Figures and Tables

**Table 1:** Statistics for the dataset according to the number of enzymes and their relationships

	enzyme	relation
# of total occurrences	35,134	17,567
# of unique elements	218	1,275
max # per organism	544	123
min # per organism	46	26
avg # per organism	68	217
stdev across organisms	26	133

**Table 2:** The nine reference pathways used in the analysis

MAP No. (KEGG)	pathway name
00010	glycolysis/gluconeogenesis
00020	citrate cycle (TCA cycle)
00030	pentose phosphate pathway
00051	fructose and mannose metabolism
00052	galactose metabolism
00620	pyruvate metabolism
00630	glyoxylate and dicarboxylate metabolism
00640	propanoate metabolism
00650	butanoate metabolism

**Table 3:** The 81 organisms included in the phylogenetic analysis. Full scientific names were abbreviated into three character notation (Abbr.) and their domain informations in phylogeny were also represented in single character that are Eubacteria (B), Archaea (A) and Eukaryota (E), respectively.

Abbr.	Domain	Organism	Abbr.	Domain	Organism
Aae	B	*Aquifex aeolicus*	Mth	A	*Methanobacterium thermoautotrophicum*
Ana	B	*Anabaena *sp.	Mtu	B	*Mycobacterium tuberculosis *H37Rv
Atc	B	*Agrobacterium tumefaciens *C58 Cereon	Nma	B	*Neisseria meningitidis *serogroup A
Ath	E	*Arabidopsis thaliana*	Nme	B	*Neisseria meningitidis *serogroup B
Atu	B	*Agrobacterium tumefaciens *C58 UWash	Oih	B	*Oceanobacillus iheyensis*
Bha	B	*Bacillus halodurans*	Pab	A	*Pyrococcus abyssi*
Bme	B	*Brucella melitensis*	Pae	B	*Pseudomonas aeruginosa*
Bsu	B	*Bacillus subtilis*	Pai	A	*Pyrobaculum aerophilum*
Cac	B	*Clostridium acetobutylicum*	Pfu	A	*Pyrococcus furiosus*
Ccr	B	*Caulobacter crescentus*	Pho	A	*Pyrococcus horikoshii*
Cel	E	*Caenorhabditis elegans*	Pmu	B	*Pasteurella multocida*
Cje	B	*Campylobacter jejuni*	Rno	E	*Rattus norvegicus*
Cmu	B	*Chlamydia muridarum*	Rso	B	*Ralstonia solanacearum*
Cpa	B	*Chlamydophila pneumoniae *AR39	Sam	B	*Staphylococcus aureus *MW2
Cpe	B	*Clostridium perfringens*	Sau	B	*Staphylococcus aureus *N315
Cpj	B	*Chlamydophila pneumoniae *J138	Sav	B	*Staphylococcus aureus *Mu50
Cpn	B	*Chlamydophila pneumoniae *CWL029	Sce	E	*Saccharomyces cerevisiae*
Cte	B	*Chlorobium tepidum*	Sco	B	*Streptomyces coelicolor*
Ctr	B	*Chlamydia trachomatis*	Sme	B	*Sinorhizobium meliloti*
Dme	E	*Drosophila melanogaster*	Spg	B	*Streptococcus pyogenes *M3
Dra	B	*Deinococcus radiodurans*	Spm	B	*Streptococcus pyogenes *M18
Ece	B	*Escherichia coli *O157 EDL933	Spo	E	*Schizosaccharomyces pombe*
Ecj	B	*Escherichia coli *K-12 W3110	Spy	B	*Streptococcus pyogenes*
Eco	B	*Escherichia coli *K-12 MG1655	Sso	A	*Sulfolobus solfataricus*
Ecs	B	*Escherichia coli *O157 Sakai	Stm	B	*Salmonella typhimurium*
Fnu	B	*Fusobacterium nucleatum*	Sto	A	*Sulfolobus tokodaii*
Hal	A	*Halobacterium *sp.	Sty	B	*Salmonella typhi*
Hin	B	*Haemophilus influenzae*	Syn	B	*Synechocystis *sp.
Hpj	B	*Helicobacter pylori *J99	Tac	A	*Thermoplasma acidophilum*
Hpy	B	*Helicobacter pylori *26695	Tel	B	*Thermosynechococcus elongatus*
Hsa	E	*Homo sapiens*	Tma	B	*Thermotoga maritima*
Lin	B	*Listeria innocua*	Tpa	B	*Treponema pallidum*
Lla	B	*Lactococcus lactis*	Tte	B	*Thermoanaerobacter tengcongensis*
Lmo	B	*Listeria monocytogenes*	Tvo	A	*Thermoplasma volcanium*
Mac	A	*Methanosarcina acetivorans*	Vch	B	*Vibrio cholerae*
Mja	A	*Methanococcus jannaschii*	Xax	B	*Xanthomonas axonopodis*
Mle	B	*Mycobacterium leprae*	Xca	B	*Xanthomonas campestris*
Mlo	B	*Mesorhizobium loti*	Xfa	B	*Xylella fastidiosa*
Mma	A	*Methanosarcina mazei*	Ype	B	*Yersinia pestis*
Mmu	E	*Mus musculus*	Ypk	B	*Yersinia pestis *KIM
Mtc	B	*Mycobacterium tuberculosis *CDC1551			

**Table 4:** Comparison of similarity scores with respect to NCBI taxonomy for 65 organisms with the glycolysis pathway (*β *= 0.8).

Method	Similarity score
Our method	0.196
[11]	0.154
